# Supplementary material for: Potential UV-Protective Effect of Freestanding Biodegradable Nanosheet-Based Sunscreen Preparations in XPA-Deficient Mice
Source: Pharmaceutics. 2022 Feb 17;14(2):431. doi: 10.3390/pharmaceutics14020431 (PMC8878169; doi:10.3390/pharmaceutics14020431)
Supplement: Supplementary file 1 [file pharmaceutics-14-00431-s001.zip › pharmaceutics-1579577-supplementary.pdf]

pharmaceutics

MDPI

Supplementary Materials: Potential UV-Protective Effect of Freestanding Biodegradable Nanosheet-Based Sunscreen Preparations in XPA-Deficient Mice

Tomomi Hatanaka, Khampeeraphan Ramphai, Shun Takimoto, Hiromi Kanda, Nami Motosugi, Minoru Kimura, Tomotaka Mabuchi, Midori Oyama, Tomoharu Takeuchi and Yosuke Okamura

Ultraviolet visible light absorption (UV–Vis) spectroscopy of nanosheet preparations and commercial sunscreen products.

To choose a suitable UV absorber for poly (L-lactic acid) (PLLA) nanosheet preparations, some fabrication trials were performed using several UV absorbers through a spin-coating-assisted layer-by-layer method using poly (vinyl alcohol) (PVA) membrane as a sacrificial layer [1–3]. Briefly, a sacrificial layer was made by spin-coating a substrate with 1.0% PVA aqueous solution, dried and spin-coated with a dichloromethane solution containing 1.0% PLLA and 0.5% a UV absorber. The sacrificial layer was removed by immersion in water, and a trial product was obtained after drying. The UV–Vis spectrums of trial products were measured with a UV spectrophotometer UV-2600 (Shimadzu, Kyoto, Japan) by adhering to a quartz plate having thickness of 2.0 mm and compared with those of commercial sunscreen products, which were applied to the plate at 2.0 mg/cm^2 [4].

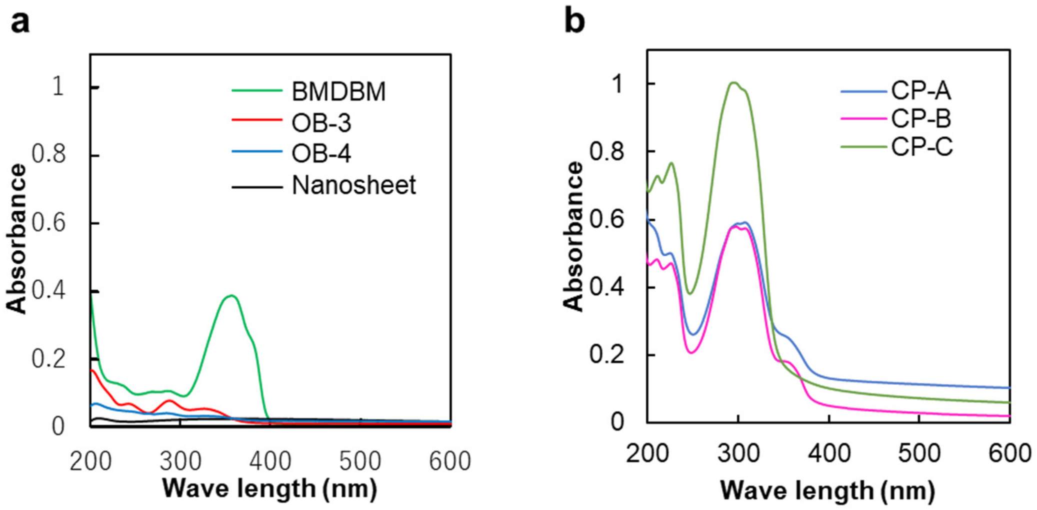

Figure S1. Ultraviolet visible light absorption spectra of nanosheet preparations and commercial sunscreen products. (**a**) Nanosheet preparations with and without avobenzone (BMDBM), oxybenzone-3 (OB-3) and oxybenzone-4 (OB-4). (**b**) Commercial sunscreen products (CP) A, B and C.

References

1. Fujie, T.; Okamura, Y.; Takeoka, S. Ubiquitous Transference of a free-standing polysaccharide nanosheet with the development of a nano-adhesive plaster. *Adv. Mater.* **2007**, 19, 3549-3553.
2. Okamura, Y.; Kabata, K.; Kinoshita, M.; Saitoh, D.; Takeoka, S. Free-standing biodegradable poly(lactic acid) nanosheet for sealing operations in surgery. *Adv. Mater.* 2009, 21, 4388-4392.
3. Hatanaka, T.; Saito, T.; Fukushima, T.; Todo, H.; Sugibayashi, K.; Umehara, S.; Takeuchi, T.; Okamura, Y. Potential of biocompatible polymeric ultra-thin films, nanosheets, as topical and transdermal drug delivery devices. *Int. J. Pharm.* **2019**, 565, 41-49.
4. Petersen, B.; Wulf, H.C. Application of sunscreen-theory and reality. *Photodermatol. Photoimmuno.l Photomed.* **2014**, 30, 96-101.
